# Multi-Species Phylogeography of Arid-Zone Sminthopsinae (Marsupialia: Dasyuridae) Reveals Evidence of Refugia and Population Expansion in Response to Quaternary Change

**DOI:** 10.3390/genes11090963

**Published:** 2020-08-20

**Authors:** Linette S. Umbrello, Raphael K. Didham, Ric A. How, Joel A. Huey

**Affiliations:** 1 School of Biological Sciences, University of Western Australia, 35 Stirling Highway, Perth, WA 6009, Australia; Raphael.Didham@csiro.au (R.K.D.); joel.huey@museum.wa.gov.au (J.A.H.); 2 Western Australian Museum, Locked Bag 49, Welshpool DC, WA 6986, Australia; ric.how@uwa.edu.au; 3 CSIRO Health & Biosecurity, Centre for Environment and Life Sciences, Floreat, WA 6014, Australia; 4 School of Human Science, University of Western Australia, 35 Stirling Highway, Perth, WA 6009, Australia; 5 Biologic Environmental Survey, 24 Wickham Street, East Perth, WA 6004, Australia

**Keywords:** Pilbara, desert, *Planigale*, *Sminthopsis*, Pleistocene, population expansion, refugia, Australia, D-loop

## Abstract

Historical population contraction and expansion events associated with Pleistocene climate change are important drivers of intraspecific population structure in Australian arid-zone species. We compared phylogeographic patterns among arid-adapted Dasyuridae (*Sminthopsis* and *Planigale*) with close phylogenetic relationships and similar ecological roles to investigate the drivers of phylogeographic structuring and the importance of historical refugia. We generated haplotype networks for two mitochondrial (control region and cytochrome b) and one nuclear (omega-globin) gene from samples distributed across each species range. We used Φ_ST_ to test for a genetic population structure associated with the four Pilbara subregions, and we used expansion statistics and Bayesian coalescent skyline analysis to test for signals of historical population expansion and the timing of such events. Significant population structure associated with the Pilbara and subregions was detected in the mitochondrial data for most species, but not with the nuclear data. Evidence of population expansion was detected for all species, and it likely began during the mid-late Pleistocene. The timing of population expansion suggests that these species responded favorably to the increased availability of arid habitats during the mid-late Pleistocene, which is when previously patchy habitats became more widespread. We interpret our results to indicate that the Pilbara region could have acted as a refugium for small dasyurids.

## 1. Introduction

In the northern hemisphere, environmental change during the Pleistocene elicited range contraction into southern refugia during repeated glacial maxima (GM) [1], or, expansion in the case of cold-tolerant species [2]. In non-glaciated regions, the absence of widespread ice-sheets has led to idiosyncratic rather than leading-edge phylogeographic patterns, resulting in multiple, species-specific refugia distributed in climatically altered landscapes [3]. In arid regions, habitat shifts were associated with the activation of mobile dune systems as vegetation patterns accommodated climate change and resulted in a complex array of species responses to change during the Quaternary [4], including in Australian deserts [5,6].

Over 75% of the Australian continent falls within arid climate zones [7]. The onset of aridification within Australia coincided with the formation of the Antarctic Circumpolar Current around the Eocene/Oligocene boundary [8] as the continent began a northward drift toward the equator, and previously widespread forest habitat declined during the Miocene [5,9]. Global Pleistocene glacial cycles resulted in extremely low levels of precipitation and sea levels during GM, most recently the last GM (approximately 15–25 kya), exacerbating the aridification of Australia’s center while forest habitats contracted to the coastal fringes [10]. Low precipitation rates and high wind erosion during repeated GM resulted in the formation of vast mobile dune systems from the mid-late Pleistocene [11]. Active dune systems were likely inhospitable to terrestrial species, which persisted in evolutionary refugia, i.e., topographically complex regions that were more climatically stable, such as inland ranges [12,13]. Then, species re-colonized habitats outside of refugia as conditions became more favorable and post-GM range expansion resulted in less diverse and younger lineage age for populations outside refugia [14]. In contrast, other species experienced range expansion during GM as heightened aridity caused their preferred habitats to shift. This explanation has been proposed for several arid-adapted bird species that retain signals of historical hybridization and introgression [6,15]. In arid Australia, intraspecific population structure results from repeated population contraction and expansion events that occurred throughout the Pleistocene from multiple localized refugia. These cyclical processes in the arid zone tend to produce species-specific responses due to varying habitat and ecological requirements within different lineages, compared to widespread southern and northern contraction among Northern hemisphere species during glaciation cycles [16].

The Pilbara is a large bioregion (approximately 179,000 km^2^) [17] in the northwest of Western Australia that is recognized as an evolutionary refugium and biodiversity hotspot in the arid zone [18,19]. It is a major geological unit that exhibits a wide range of landforms that are divided into four distinct subregions under the Interim Biogeographic Regionalization for Australia (IBRA) based on shared climate, geology, and vegetation characteristics [20]. These subregions include the sandy coastal plains of the Roebourne, the granite and greenstone terrain of the Chichester (which includes several range systems and plains), the alluvial plains of the Fortescue (including the Fortescue River), and the sedimentary and volcanic Hamersley Range (that is rich in banded ironstone formations) of the Hamersley [17]. The Pilbara’s geological complexity, leading to landscape and substrate heterogeneity, has resulted in a diversity of available habitats across the region that have acted as evolutionary refugia for many rock- and mesic-adapted species [21,22].

Phylogeographic patterns within the Pilbara are closely associated with substrate types, particularly rocky habitats, and many Pilbara-endemic species show limited dispersal into the adjacent sandy desert regions surrounding it to the north and east [22,23]. Within the Pilbara, the discontinuity of substrate types across the Chichester–Fortescue–Hamersley IBRA subregion boundaries has been implicated in driving population structure in rock-specialized geckos [22], a pebble-mimic dragon *Tympanocryptis cephalus* [24], and hummock (*Triodia* species) grasses [23]. The underlying geology, Fortescue River valley, and IBRA subregion boundaries have all been proposed as important landscape features that drive the genetic population structure [22]. However, these ideas have mostly been derived from species with limited dispersal abilities and strict habitat preferences; they have not been examined in species with higher dispersal ability, and broader habitat preferences that were exposed to Pleistocene induced changes across the Pilbara.

One arid-adapted group that remains relatively widespread and abundant in arid habitats compared to other mammal groups [25] is the dasyurid marsupials, which diversified during the Miocene [26,27]. Their success can be linked to remarkable adaptations allowing persistence in arid environments, such as high dispersal abilities [28], extended breeding seasons (6–8 months) with polyoestry and multiple offspring (6–10) [29], and physiological adaptations, such as caudal fat storage and daily torpor, which reduce energy requirements and water loss [30,31]. Some 30 dasyurid species occur in arid habitats, and the genera *Sminthopsis*, *Planigale,* and *Ningaui* (subfamily Sminthopsinae) are highly diverse with over 20 species [32]. Species in the Sminthopsinae are particularly suitable for comparative phylogeography as they have extensive distributions across a variety of arid habitats, allowing for comparisons across multiple bioregions and habitat types.

In this study, we explore the processes that generate phylogeographic structuring in arid occurring dasyurids, with a particular focus on the role of the Pilbara as an evolutionary refugium. We selected six co-distributed species of *Sminthopsis* and *Planigale* that occur in the Pilbara that have shared evolutionary histories and similar life history strategies, but different dispersal potential and habitat preferences. Using DNA sequence data to test for restricted gene flow across the boundary of the Pilbara, and between the IBRA subregions within the Pilbara, we expected higher levels of genetic diversity to occur within the Pilbara if that region had acted as an historic refugium for small dasyurids. Additionally, we test for historical demographic responses to past environmental change within this confamilial group to identify responses driven by GM habitat contractions between species with different dispersal abilities and divergent habitat specificities, particularly since the last GM.

## 2. Materials and Methods

### 2.1. Study Species

**Figure 1:**
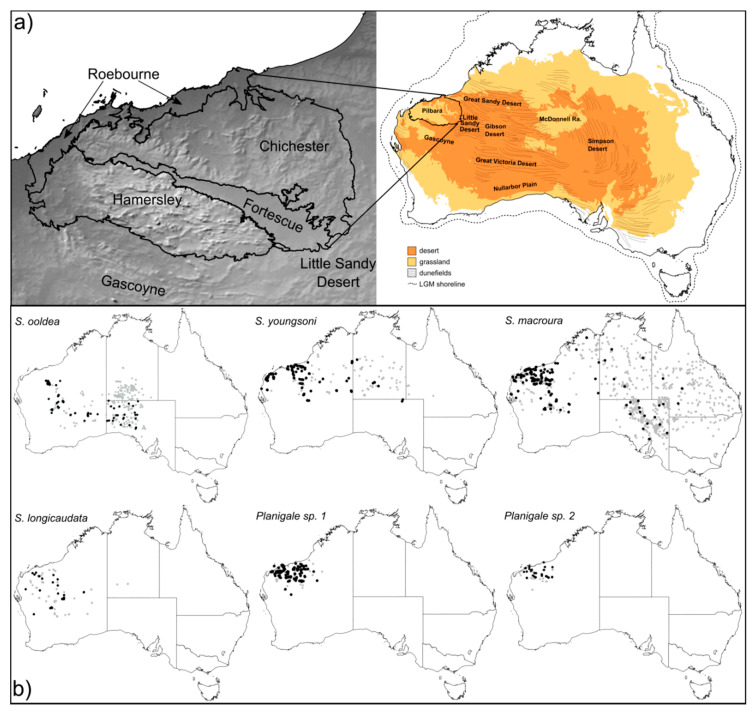
(**a**) Map of Australia showing arid regions labeled as Desert and Grassland (see [7]), location and direction of major dune systems [41], approximate location of Last Glacial Maxima (LGM) shoreline [5], and the Pilbara bioregion outlined in black, inset map shows digital elevation model and detail of Interim Biogeographic Regionalization for Australia (IBRA) within the Pilbara. (**b**) Distribution maps of the six species (gray points indicate vouchered specimens in Australian museums), and tissues sequenced in this study (black points). Non-WA records were accessed from Atlas of Living Australia.

Six small dasyurid species (subfamily Sminthopsinae), *Sminthopsis longicaudata* [33], *Sminthopsis macroura* [34], *Sminthopsis ooldea* [35], *Sminthopsis youngsoni* [36], *Planigale* sp. 1, and *Planigale* sp. 2, were selected for this study. The two unnamed *Planigale* species have previously been determined from molecular data [37] and are morphologically distinct (K.P. Aplin pers. comm.). As it is currently recognized, S. macroura represents a species complex with potentially five named taxa that were all synonymized by Archer [38]. Molecular data have shown that at least three distinct species (*S. macroura*, *S. froggatti*, and *S. stalkeri*) should be recognized, as well three lineages within the ‘true’ *S. macroura*, one of which occurs in the northwest of Western Australia (WA) [39] and is somewhat geographically isolated (Figure 1b). The study species each have different habitat preferences and exhibit a range of potential dispersal abilities (Table 1). Both Planigale species are endemic to the greater Pilbara region [40], while the four Sminthopsis occur in the Pilbara and also in the adjacent arid regions (Figure 1b).

### 2.2. DNA Extraction and Amplification and Sequence Alignment

Total genomic DNA was obtained from 833 samples held at the WA Museum, the WA Department of Biodiversity, Conservation and Attractions, and the Australian Biological Tissue Collection at the South Australian Museum (see Table S1 Supplementary Materials). Biological material was preserved in 70–100% ethanol or cryo-frozen at −75 °C, and it included liver, muscle, heart, ear, tail, and toe. For DNA extractions, approximately 5–25 mg of biological material was used in Qiagen DNeasy tissue and blood kits according to the manufacturers’ instructions, and DNA was eluted into 60–120 µL tris buffer. Several mitochondrial and nuclear loci were screened for suitable degrees of intraspecific variation across the study species and control region (*CR*), cytochrome b (*cytb*), and omega globin (*ω-globin*) were chosen, GenBank accession numbers, including those for non-suitable loci sequences are in Table S1 (MT366215–MT454794). Primers used for *CR*, *cytb,* and *ω-globin* are listed in Table S2.

All regions were amplified via polymerase chain reaction (PCR) as described in [42], following the PCR cycling conditions of [43]. For the amplification of *CR* for *S. youngsoni*, the annealing temperature was increased from 50 to 57 °C due to the presence of large repeat regions at the 5′ end (see [44]). DNA purification and bidirectional sequencing was carried out at the Australian Genome Research Facility, Perth WA. Forward-reverse assemblies, quality control, and alignment of sequences was performed in GENEIOUS v.9.1.7 [45], as described in [42]. Final sequence length for *CR* ranged from 560 to 800 bp, and *ω-globin* ranged from 770 to 790 bp (Table 2). Only approximately the first 1000 bp of *cytb* could be amplified for *Planigale* sp. 1, *Planigale* sp. 2, and WA *S. macroura* samples.

### 2.3. Species/Lineage Boundaries in S. macroura

**Figure 2:**
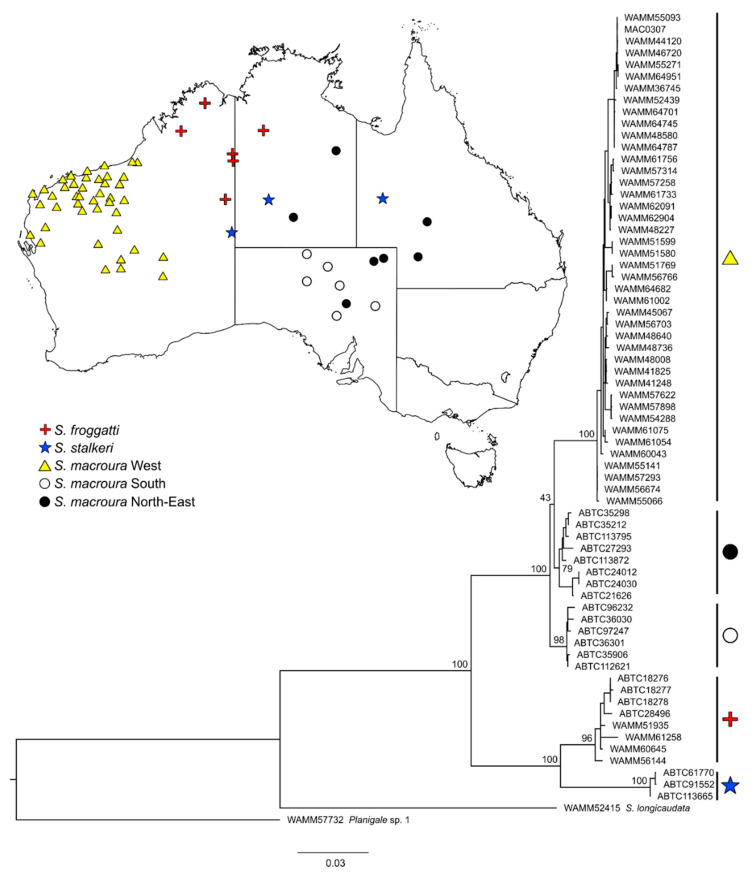
Maximum likelihood tree of *Sminthopsis macroura* complex using concatenated (control region, cytochrome b, and omega globin) sequence data. Bootstrap support for major nodes is shown. Names of *S. macroura* populations follow Blacket et al. [39].

To determine the geographic extent of the WA lineage of *S. macroura*, previously identified from three samples by Blacket et al. [39], a maximum likelihood tree was built on the concatenated DNA sequences of a subset of *S. macroura* samples using the GENEIOUS RAXML plug-in [46]. Two additional samples, *Planigale* sp. 1 (WAM M57732) and *S. longicaudata* (WAM M52415), were used as outgroups. The model selecting program PARTITIONFINDER v. 1.1.1 [47] was used and selected GTR + G, with the alignment partitioned by gene and codon position for *cytb*, and run for 1000 bootstrap replicates. Based on the tree analysis (Figure 2), only the WA lineage was considered for the subsequent phylogeographic and population-level analysis.

### 2.4. Phylogeographic Structure Analysis

**Figure 3:**
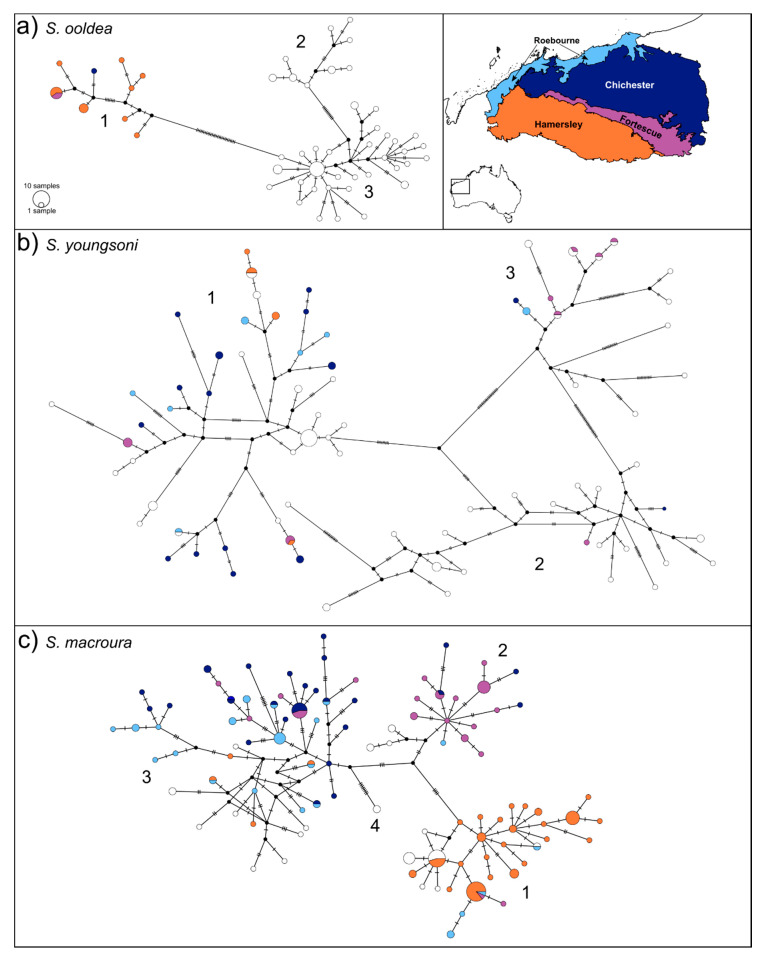

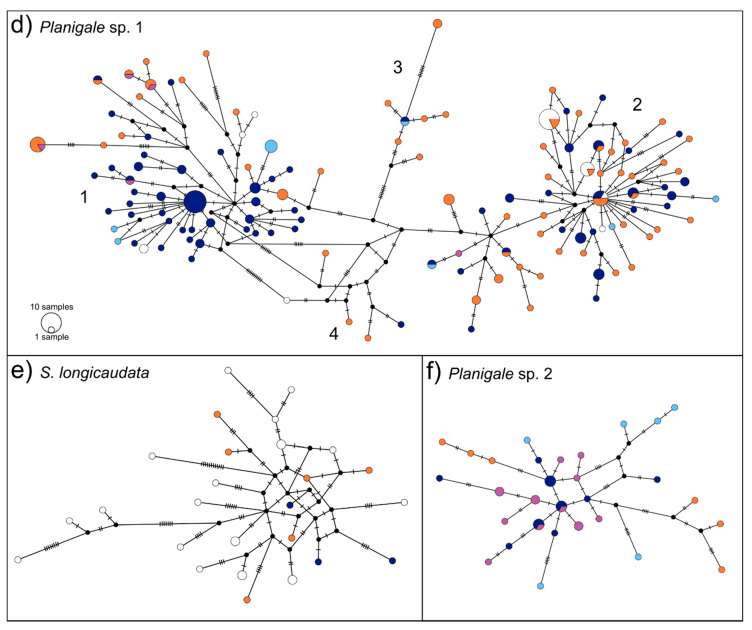
Concatenated mtDNA networks and map of samples for (**a**) *S. ooldea* (*n* = 70), (**b**) *S. youngsoni* (*n* = 113), (**c**) *S. macroura* (*n* = 177), (**d**) *Planigale* sp. 1 (*n* = 206), (**e**) *S. longicaudata* (*n* = 30), and (**f**) *Planigale* sp. 2 (*n* = 39). Circle size indicates the number of individuals sharing that haplotype, with larger circles indicating more common haplotypes, small black circles indicating nodes, and hatch marks showing missing haplotypes. Haplotypes are colored by the four IBRA regions and white for ‘non-Pilbara’ samples (see map top right). The major haplo groups are numbered. Concatenated mtDNA networks and map of samples for.

To investigate phylogeographic patterns, TCS neighborhood-joining haplotype networks were built using the software program POPART [48]. POPART excludes gaps and ambiguous bases from the analysis, so alignments with difficult to resolve indel regions (e.g., the 5′ end of *CR*) were cleaned using the online program GBLOCKS v.0.19b, using default settings [49] (available through the website phylogeny.fr; [50]). Nuclear sequences were phased using the statistical haplotyping program PHASE [51], and input files were created using the online web-tool seqPHASE (http://seqphase.mpg.de/seqphase/; [52]) where the allele combinations with probability scores above 0.7 were chosen for the analysis (for *S. ooldea* and *S. longicaudata*, we allowed scores from 0.5 to be included to ensure all variation was captured). The final length of the cleaned and trimmed alignments used for analysis is given in Table 2 as L. Then, statistical parsimony TCS networks [53] were built for each loci and the concatenated mtDNA alignments of each species (Figure 3).

### 2.5. A Priori Groupings for Geographical Analysis

We tested for significant population structure associated with the Pilbara region and major subregions within the Pilbara among the study species. The IBRA subregions contain distinct geology, landscape features, and vegetation that are important for determining species occurrences and shaping genetic structure [22]. To calculate the equilibrium genetic differentiation summary statistics, such as Φ_ST_, we clustered individuals that were continuously distributed across the landscape into subpopulations. To test for population differentiation across the Pilbara boundary, we grouped each species into “Pilbara” (i.e., those samples that fell within the Pilbara bioregion), and “non-Pilbara” populations (i.e., those outside of the Pilbara). This resulted in two populations, except for *S. youngsoni*, which had three populations, as the distribution of this species is disjunct across the south of the Pilbara (Figure 1b). The two *Planigale* species were excluded from this level of analysis due to low sample numbers outside of the Pilbara. To test for population differentiation within the Pilbara, samples were grouped into subpopulations based on the four IBRA subregions—Roebourne, Chichester, Fortescue, and Hamersley (Figure 1a), as these subdivisions capture the geographic complexity of the two major range systems and the low-lying Fortescue river valley, which could impact the dispersal of small mammals. *S. ooldea* and *S. longicaudata* were not included in this level of analysis, due to low numbers of samples within the Pilbara that were predominantly found in just one IBRA subregion. Then, these aforementioned groupings were used to run global Φ_ST_ and pairwise Φ_ST_ tests on the *cytb* alignments for each species (only *cytb* was used due to the low coverage of *CR* for *S. longicaudata*) in the program ARLEQUIN v.3.5.2.2 [54]. To account for different sample sizes between subpopulations, we also used a rarefaction approach to calculate the Haplotypic Richness and the number of Private Haplotypes as implemented in the program HP-Rare [55].

### 2.6. Tests for Historical Population Size Changes

**Figure 4:**
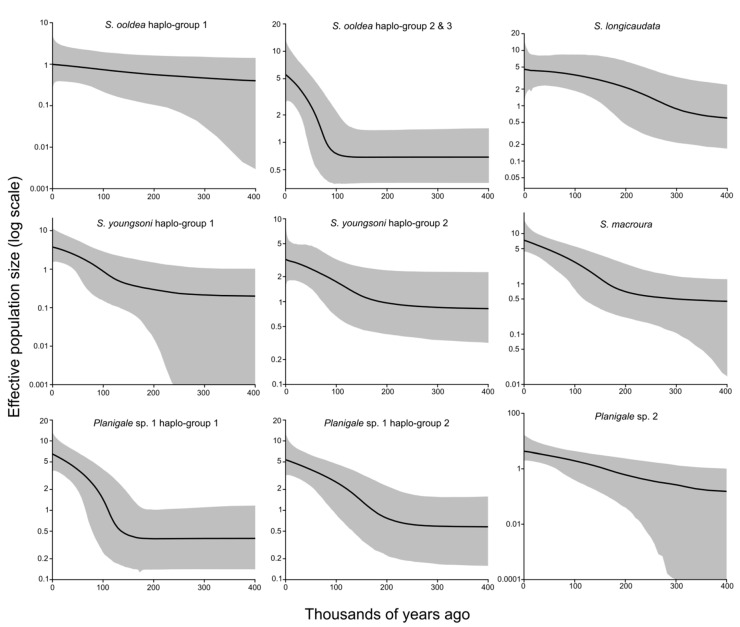
Extended Bayesian skyline plots showing population size changes over time using the combined mitochondrial (*CR* + *cytb*) and nuclear DNA sequence data of each species and haplo group in this study. The median posterior estimates of demographic change over the past 400,000 years are graphed with the gray area indicating the 95% central posterior density/credible intervals.

We tested for evidence of historical population expansion among the study species and subpopulations. Molecular variation indices and expansion statistics were calculated in DNASP v.5 [56] on the concatenated mtDNA and nDNA alignments used in the network analysis for each species, or haplo group where appropriate based on the network results. Significance values for Ramos-Onsins R2 and Fu’s Fs were obtained under coalescent simulations with segregating sites. Rates and timing of population expansion were investigated using the extended Bayesian skyline model (EBSP) [57] in BEAST2 [58]. Skyline analysis assumes that sampling is from a panmictic population [59]; so, where species had structured populations, identified from the mtDNA network analysis, these were analyzed separately, and only those that successfully converged are shown (Figure 4). PARTITIONFINDER was used to estimate substitution models for each of the three genes and codons for *cytb*; however, most models failed to converge when *cytb* was partitioned by codon, so it was not partitioned for any analyses except for *S. ooldea*. Site models from PARTITIONFINDER were selected, with the exception of estimating the portion of invariable sites (+I), as it can overcomplicate the model and is not considered biologically meaningful for intraspecific level data [60]. For *Sminthopsis* species, a substitution rate of 0.0105 substitutions/site/Myr and standard deviation (sd) of 0.001 using a normal prior, and a rate of 0.0095 substitutions/site/Myr (sd of 0.0005) for *Planigale* species were used to capture the ranges in Krajewski et al. [61], who reported different *cytb* mutation rates for each genus. A uniform prior was used for *CR* and *ω-globin* with substitution rates estimated. The EBSP analysis was run for 100 million generations to ensure that the effective sample sizes of posterior parameter estimates were ≥ 200. The EBSP log files were plotted in R using the ‘plotEBSP.R’ script [57].

## 3. Results

### 3.1. Relationships within the Sminthopsis macroura Complex

Tree analysis on *S. macroura* samples supported the distinctiveness of a WA lineage (bootstrap support 100%) and indicated that the distribution of the WA lineage is restricted to northwest and central WA (Figure 2). Subsequent downstream analysis focused only on the WA lineage.

### 3.2. Gene Diversity and Haplotype Distribution

All six species had multiple haplotypes (30–130) and high haplotype diversity (0.88–0.99) for the concatenated mtDNA dataset (Table 2). Haplotype richness and the number of private haplotypes under rarefaction were highly correlated (correlation coefficient = 0.99) and were lower for the Pilbara compared to non-Pilbara samples for *S. ooldea*, *S. longicaudata,* and *S. youngsoni*, and higher within the Pilbara for *S. macroura* (Table 2). Haplotypes that formed clades in the network analysis were grouped together and hereafter referred to as ‘haplo groups’ (Figure 3). Allele diversity for the phased nuclear data was lower than the mtDNA loci and did not correlate to geographic regions in the network analysis (see Figure S1, Supplementary Material).

The restriction of haplo groups to geographic regions occurred in the mtDNA sequence data for *S. ooldea*, *S. macroura*, and slightly for *Planigale* sp. 1, but no obvious geographically restricted haplo groups were detected for *S. longicaudata*, *S. youngsoni,* or *Planigale* sp. 2. Of the 50 mtDNA haplotypes for *S. ooldea*, nine were restricted to the Pilbara (halpo group 1) and separated by 28 mutations from the other samples (Figure 3a). There were 76 mtDNA haplotypes for *S. youngsoni*, of which 25 only occurred in the Pilbara, and the species had three haplo groups; two of these were separated by 15 mutations and occurred on either side of the Gibson Desert (halpo group 1 and 2; Figure S2), and a third group that was 30 mutations from haplo groups 1 and 2 was distributed throughout WA (haplo group 3, Figure 3b). The WA lineage of *S. macroura* had 75 of the 100 mtDNA haplotypes located in the Pilbara only, and four haplo groups were identified, though with fewer mutations between each when compared to *S. ooldea* and *S. youngsoni*. Of these, haplo group 1 was mostly restricted to the Hamersley subregion; by comparison, the most diverse haplo group (3) had few individuals occurring within the Hamersley, instead being distributed widely throughout the Chichester and Carnarvon regions (Figure 3c; Figure S2). *Planigale* sp. 1 exhibited four haplo groups (Figure 3d), the larger two (1 and 2) being split across the center of the Pilbara with haplo group 2 occupying the eastern half of the Chichester and Fortescue subregions, which was exclusive to the other halpo groups (Figure S2). S. *longicaudata* had 26 haplotypes, of which nine were only found in the Pilbara, most of which were distributed throughout the network (Figure 3e). *Planigale* sp. 2 had lower nucleotide diversity for all three genes compared to the other five species (Table 2), and the phylogeographic structure in the network analysis did not correlate to the IBRA subregions (Figure 3f).

### 3.3. Pilbara Phylogeographic Analysis

Analysis of population differentiation (Φ_ST_) across the Pilbara boundary revealed significant differentiation within *S. ooldea* (Φ_ST_ = 0.785, *p* < 0.001), *S. youngsoni* (global Φ_ST_ = 0.225, *p* < 0.001; Table 3), and *S. macroura* (Φ_ST_ = 0.0284, *p* = 0.012), but not *S. longicaudata* (Φ_ST_ = 0.026, *p* = 0.168). Within the Pilbara population, structure was tested between the four IBRA subregions (Figure 1a), and global Φ_ST_ values were significant for all four species: *S. macroura* (Φ_ST_ = 0.398 *p* < 0.001), *Planigale* sp. 2 (Φ_ST_ = 0.273, *p* < 0.001), *S. youngsoni* (Φ_ST_ = 0.206, *p* < 0.001), and *Planigale* sp. 1 (Φ_ST_ = 0.075, *p* < 0.001). Most pairwise Φ_ST_ tests on the subpopulations between the four Pilbara IBRA subregions showed significant subpopulation differentiation except between Chichester and Roebourne for *S. youngsoni* and *Planigale* sp. 1, and between Chichester and Hamersley for *Planigale* sp. 2 (Table 3).

### 3.4. Population Expansion Analysis

Population size changes were tested using summary statistics (Table 4) and extended Bayesian skyline analysis (Figure 4). Population expansion was detected for the concatenated mtDNA sequence data for all six species and subpopulations with significant and high Fu’s *Fs* values; however, test scores for Tajima’s *D* and Ramos-Onsins *R2* were not significant except for *R2* for *Planigale* sp. 1 (Table 4). There was almost no evidence of population expansion with the nuclear loci, except for *S. youngsoni*, which was significant for *R2* and *Fs*. Coalescent skyline analyses showed population size increases for all haplo groups examined except *S. ooldea* haplo group 1 (Figure 4). The onset of population expansion occurred between 150 and 200 kya for *S. youngsoni*, *S. macroura*, and *Planigale* sp. 1, and 100 kya for *S. ooldea*. Expansion began earlier for *S. longicaudata,* approximately 330 kya, and *Planigale* sp. 2 showed a steady increase in size, but large credibility intervals indicate uncertainty with population size estimates earlier than 200 kya.

## 4. Discussion

Late Quaternary climate changes were a major driver of species phylogeographic structure throughout arid Australia, resulting in intraspecific variation from multiple population expansion and contraction events across an array of species [5]. Here, we show that the demographic responses among related dasyurid species to past climate change exhibit similar patterns of population expansion during the Pleistocene, suggesting that their shared evolutionary history and adaptation to a climatically unstable and relatively young landscapes has determined patterns of genetic structure in these species.

### 4.1. Pilbara Refugia

The Pilbara is recognized as a biodiversity hotspot [18,22] and is home to a number of endemic mammal species [40]. We hypothesized that there would be higher genetic diversity within Pilbara populations if the region had acted as a refugium for small dasyurids during GM. However, mtDNA haplotypic richness was only markedly higher in the Pilbara for *S. macroura*, among the four congeneric species tested, when compared to populations outside the Pilbara. This indicates that the Pilbara may have acted as an evolutionary refugia for the WA lineage of *S. macroura* in the past, but that other regions within the distributions of *S. ooldea*, *S. longicaudata,* and *S. youngsoni* have been equally as, if not more, important as the Pilbara in preserving haplotype diversity over time. What is more significant, as shown by our results, is the impact of the Pilbara geological boundary as a barrier to gene flow for small dasyurids. We show significant population differentiation associated with that boundary in the majority of species, indicating that most species do not readily disperse across the Pilbara boundary, which is likely due to the discontinuity of habitats over this boundary [22].

We have identified the southeastern edge of the Pilbara craton as an important phylogeographic break between Pilbara and non-Pilbara samples of *S. ooldea*, despite this species occurring continuously from the Pilbara into central South Australia (Figure 1b). This differentiation may be attributed to the disruption of the *Acacia*-dominated habitat between the edge of the Pilbara craton and the sandy substrates of the adjacent Little Sandy Desert, since *S. ooldea* prefers *Acacia* woodland habitats [62]. The disjunction between the rocky Pilbara craton and the dune systems of the Great and Little Sandy Deserts has been identified as a limiting factor in the dispersal of non-sand-preferring species [63], such as the gecko *Lucasium stenodactylum*, where genetically well-defined clades occur in close proximity at the southeastern edge of the Pilbara Craton [64].

The Pilbara has been proposed as an important Pleistocene refugium during the heightened arid conditions of the last 400,000 years [5,22]. During this time, the topographically complex region was isolated by the surrounding lowlands through the formation of vast sand dune deserts [65] that restricted the range and dispersal of species adapted to the older rocky, clay, or stony substrates, and facilitated the range expansion of species preferring sandy habitats [14,23]. The differentiation of subpopulations across the Pilbara boundary of *S. youngsoni*, *S. macroura,* and *S. longicaudata* was less distinct in the haplotype networks than *S. ooldea*, but it still produced significant Φ_ST_ results, suggesting greater dispersal or habitat connectivity for these three species. While the Φ_ST_ results suggest differentiation between the Pilbara and eastern populations of *S. youngsoni*, it is probably significantly weighted by the far eastern samples, as sandy habitats are continuous to the north and east of the Pilbara region (Figure 1a).

Our data show that the southeastern Pilbara area may have contained a restricted population of *S. ooldea* in the past, as indicated in the mtDNA (but not in the nuclear DNA). The strongest evidence we found was for the Pilbara as an evolutionary refugium for widely distributed dasyurid species within the WA lineage of *S. macroura*. Haplotype diversity was higher within the Pilbara than outside of it, indicating the possible dispersal of individuals outside of the Pilbara that could be associated with a population expansion event.

### 4.2. Genetic Structure within the Pilbara

We hypothesized that species genetic structure within the Pilbara would be driven by dispersal ability, the distribution of preferred habitat types, and the location of potential biogeographic barriers. We recovered significant genetic structure between IBRA subregions of the Pilbara for *S. macroura*, *S. youngsoni*, and both *Planigale* species, with almost all pairwise Φ_ST_ comparisons being significantly different. However, the mtDNA networks revealed that the underlying patterns among the study species were not congruent. Within the Pilbara, *Planigale* sp. 1 and *S. macroura* have comparable distributions and sample sizes but exhibited different phylogeographic patterns in the mtDNA network analysis. This difference probably reflects their different habitat preferences. *Sminthopsis macroura* prefers clay/loam soils in the Pilbara lowlands, and the rocky Hamersley Range could be an important linear barrier limiting dispersal from populations in the north, thus resulting in restricted gene flow into the Hamersley subregion. A similar pattern has been observed in species of rock-specialized geckos [22], a pebble-mimic dragon *T. cephalus* [24], and hummock (*Triodia* species) grasses [23], as genetic clades mirror the discontinuity of substrate types across the Chichester–Fortescue–Hamersley IBRA subregion boundaries. In contrast, the distribution of haplo groups 1 and 2 in *Planigale* sp. 1 occurred in a broadly east–west pattern through the central Pilbara (Figure S2), and closely related haplotypes occur in the Chichester and the eastern Hamersley subregions. The clustering of clades in the eastern or western parts of the Chichester subregion was also observed in other gecko species and attributed to strong associations with habitat types and geology [22].

We did not find any clear phylogeographic structure in the mitochondrial network for *Planigale* sp. 2, but the Φ_ST_ results between the Hamersley and Fortescue IBRA subregions were significant. This could result from low levels of gene flow across a barrier, such as the mountainous Hamersley Range, leading to haplotype frequency differences, but there is also enough gene flow still occurring to interrupt the development of clear phylogeographic structure. Phylogeographic structure was not detected within the Pilbara samples of *S. longicaudata* (Figure 3e). This surprising finding is despite this species being integrally associated with rocky substrates that have been shown to drive structure in other taxa, and maybe due to low sample size (*n* = 12).

The sand specialist in this study, *S. youngsoni*, occurs in dune systems on the coast, west of the Pilbara, and throughout the expansive longitudinal dunes of the Great Sandy Desert through to the Simpson Desert in South Australia. While these habitats are relatively continuous, we recovered mtDNA population differentiation on either side of the Gibson Desert, which is a desert dominated by hard, lateritic stony plains that are unlikely to be a suitable habitat for *S. youngsoni* and probably limit its dispersal to narrow corridors along waterlines. The narrowing of sandy habitats along the northwest Pilbara coast can be seen clearly in the distribution of records of *S. youngsoni* (Figure 1), but these samples still formed a haplo group with the eastern Pilbara samples. However, the Φ_ST_ results between the Hamersley and Chichester/Fortescue subregions suggest some differentiation of populations in the east and western parts of the Pilbara region. Two other small dasyurids, *Pseudantechinus macdonnellensis* and *Sminthopsis psammophila*, which similar to *S. youngsoni* are distributed in sandy deserts, also did not exhibit any clear phylogeographic structure throughout their ranges [42,66]. The more recent formation of sand dune deserts has led to lower lineage age, intraspecific structure, and strong signals of population expansion in several gecko species [14,67] and hummock grasses [23] compared to related species occurring in more geographically structured habitats. However, we did not find significantly lower haplotype diversity in *S. youngsoni*, compared to other species in this study, suggesting that sandy habitats are just as capable of maintaining high levels of intraspecific diversity in dasyurids as other habitat types within and around the Pilbara.

We hypothesized that species occurring in restricted and patchy habitats would have stronger phylogeographic patterns than those inhabiting more extensive and continuous habitats [40]. However, this did not prove to be the case and is likely due to the high dispersal abilities of small arid-dwelling dasyurids [28]. Two species, *S. longicaudata* and *Planigale* sp. 2, that are strongly associated with isolated, patchy habitats, lacked strong population structure, despite our expectations. Both species are substrate specialists (Table 1), and they are infrequently captured on other substrate types [40]. The lack of population structure in our data could indicate that both these species have better dispersal abilities than previously documented and that population expansion has masked the signal of population structure. The lack of structure may also be a result of our data being limited in their power, having only three loci in total that includes two linked on the mitochondrial genome.

Our results add support to a growing number of studies that have found low population differentiation and potential panmixia within mammal species occurring in the Pilbara and other arid regions of Australia [66,68,69]. A recent study on three small mammals occurring in the Pilbara, two widespread habitat generalists (a murid, *Pseudomys hermansburgensis* and dasyurid, *Ningaui timealeyi*) and a pebble surface specialist mouse (*Pseudomys chapmani*) found little evidence for population structure using mitochondrial and microsatellite sequence data [68]. Levy et al. [68] proposed this resulted from the high fecundity and dispersal potential of these species, resulting in panmixia despite the complex geological landscape of the Pilbara presenting numerous potential barriers to gene flow. Panmixia has also been suggested for the mainland Pilbara population of the northern quoll [70], the largest Pilbara dasyurid (<1 kg), which is a species that has been documented to have excellent dispersal abilities [71].

### 4.3. Pleistocene Population Expansion

Byrne et al. [5] proposed that an intraspecific genetic structure may be the result of contraction and expansion from GM refugia, and that the timing of expansion should coincide with the last GM. We tested the hypothesis that population expansion among the study species likely would have occurred after the last GM. However, the timing of the onset of these expansion events we detected was much earlier than the last GM (approximately 15–25 kya), beginning instead around 100–200 kya (Figure 4). This expansion coincides with increasing aridity during peak climate oscillations of the mid-late Pleistocene and is comparable to butcherbirds [6], monsoonal/savannah occurring macropods [72], and clades of a widespread arid-adapted lineage of geckos [11], which experienced population expansion that also began during the mid-late Pleistocene. Our results are concordant with a growing number of studies that suggest population expansions of arid-adapted taxa occurred during the high amplitude climate cycles of the mid-late Pleistocene, coinciding with periods of intense aridification [14,15,23,67,73,74].

### 4.4. Conservation Implications for Arid-Occurring Dasyurids

Cryptic diversity is continuing to be a challenge in the conservation of Australia’s species, including mammal groups [37]. Our tree analysis on the *S. macroura* complex supports the differentiated mitochondrial lineages identified by Blacket et al. [39], with an added signal from an independent nuclear locus. In particular, the strength of the *S. froggatti* + *S. stalkeri* group appears to provide evidence of separate species within the current *S. macroura* group, while preliminary examination of specimens in the WA Museum (L.S. Umbrello pers. obs.) has shown that specimens genotyped as *S. froggatti* can be readily distinguished from the western lineage of *S. macroura*. However, resolution of the relationships among the *S. macroura* clades was not well supported and follows a trend from previous studies of low support for the placement of these clades [39,75]. Higher relationships among the stripe-faced dunnarts require further investigation using multiple independent nuclear loci and comparative morphology. Until these relationships are resolved, we suggest that the western lineage of *S. macroura* is categorized as a distinct Evolutionary Significant Unit [76].

## 5. Conclusions

The arid zone comprises a multitude of complex, structured habitats that have facilitated rapid adaptive radiation and niche partitioning within Dasyuridae [77,78,79,80]. The species in this study were selected based on their broadly overlapping distributions and different substrate preferences [40], and we recovered some evidence of species-specific distributions of mtDNA haplo groups. However, we also found congruence in the broader genetic patterns of small dasyurids within the Pilbara, which was likely a result of shared evolutionary history and adaptations to aridity. Small dasyurids continue to persist in Australian arid habitats despite wide-scale extinctions and declines in other arid-adapted mammals [25], and our data add to a growing number of studies finding low levels of genetic structure in widely occurring arid species (reviewed in [81]). This is in contrast to species-specific responses to Pleistocene environmental change that are driven by differences in species ecological requirements [5,16] and demonstrates the adaptive success of small dasyurids to harsh and changing environmental conditions, which allow them to persist in these environments.

## Figures and Tables

**Table 1:** Summary of attributes of the six mammal species chosen for this study (data from [29,32]).

Species	Habitat/Specializations	Reproduction	Dispersal Potential
*Sminthopsis longicaudata*	Specialist. Exposed rock/stony soils, flat-topped hills, lateritic plateaus, sandstone ranges, and breakaways. Striated foot pads for climbing rocky surfaces. Tail two times head–body length	Breed August–December, young disperse in March–April, 6 teats, polyestrous	Potentially poor, due to patchiness of preferred habitat type
*Sminthopsis macroura*	Generalist. (WA lineage) clay/loam soil. Abundant in hummock grassland where stock is absent. Shelters in cracks in soil, under rocks, logs, or burrows of other animals	Two litters from June–February 11 day gestation 8 teats	Good, quickly colonizes areas after rainfall and can move 0.3–2 km in a night
*Sminthopsis youngsoni*	Specialist. Sand dunes, inter-dune swale, red desert sand plains. Shelters in burrows often dug by lizards. Hairy feet for traversing sand	Young born in September–January, up to 6 pouch young, which are independent from November–February	Good on sandy substrates. Locally common
*Sminthopsis ooldea*	Generalist. Mulga woodlands with hummock grass understory. Less common on dunes, sandplains, clay soils, and Mallee woodland	Potentially polyestrous, young born September–January, 8 teats	Good–moderately uncommon where it occurs
*Planigale* sp. 1	Generalist. Uplands and some sandy lowlands. Hummock grasslands, rocky scree slopes, cobbled creek beds, and sandy plains	Pouch development and young in September–October 8 teats (pers. obs.)	Potentially good as common where they widely occur
*Planigale* sp. 2	Specialist. Exclusively on cracking clay soils, avoiding rocky habitats. Extremely depressed cranium for squeezing in fissures of cracking clay	Pouch development in November, 12 teats (pers. obs.)	Potentially poor, perhaps only on clay substrates

**Table 2:** Molecular diversity indices for the concatenated mtDNA (*CR* + *cytb*), and the phased nuclear (*ω-globin*) gene alignments of the six species in this study. Indices are also calculated for species within and outside of the Pilbara where appropriate. Number of samples (*n*), sequence length in base pairs excluding alignment gaps (L), number of haplotypes or alleles (Hn), haplotype diversity (*H*), nucleotide diversity (*π*), haplotypic richness under rarefaction (Hr).

Species	L	*n*	Hn	*H*	*π*	Hr	L	*n*	Hn	*H*	*π*
	Concatenated mtDNA						Phased *ω-Globin*				
*S. ooldea* (all)	1625	70	50	0.98	0.0115		774	122	14	0.86	0.0022
Pilbara		15	9	0.88	0.0045	9.0		32	6	0.52	0.0013
Non-Pilbara		55	41	0.98	0.0056	13.2		90	14	0.88	0.0022
*S. youngsoni* (all)	1923	113	76	0.99	0.0139		798	276	39	0.78	0.0022
Pilbara		43	32	0.99	0.0125	33.0		104	26	0.90	0.0004
Non-Pilbara		70	50	0.98	0.0143	33.5		172	27	0.68	0.0016
*S. macroura* (WA)	1589	177	100	0.98	0.0071		787	404	11	0.32	0.0005
Pilbara		146	84	0.98	0.0071	24.9		304	11	0.34	0.0005
Non-Pilbara		31	18	0.94	0.0067	18.0		100	5	0.29	0.0004
*S. longicaudata* (all)	1369	30	26	0.99	0.0080		777	112	13	0.78	0.0026
Pilbara		9	9	1.00	0.0057	9.0		16	7	0.82	0.0030
Non-Pilbara		21	17	0.98	0.0087	8.3		96	11	0.78	0.0026
*Planigale* sp. 1	1609	206	130	0.99	0.0078		777	432	27	0.78	0.0026
*Planigale* sp. 2	1391	39	30	0.98	0.0046		788	90	4	0.07	0.0001

**Table 3:** Pairwise Φ_ST_ estimates of population differentiation between the Pilbara and other regions of *S. youngsoni* and within the Pilbara IBRA subregions of *S. youngsoni*, *S. macroura*, *Planigale* sp. 1, and *Planigale* sp. 2 based on *cytb* DNA sequences.

Species	*n*	Subpopulations				
*S. youngsoni* outside Pilbara			Carnarvon	Pilbara	Eastern	
	26	Carnarvon	-			
	49	Pilbara	**0.147**	-		
	50	Eastern	**0.351**	**0.173**	-	
*S. youngsoni* within Pilbara			Chichester	Fortescue	Hamersley	Roebourne
	24	Chichester	-			
	9	Fortescue	**0.316**	-		
	6	Hamersley	**0.231**	**0.427**	-	
	10	Roebourne	−0.004	**0.162**	**0.129**	-
*S. macroura*			Chichester	Fortescue	Hamersley	Roebourne
	32	Chichester	-			
	23	Fortescue	**0.190**	-		
	57	Hamersley	**0.541**	**0.500**	-	
	30	Roebourne	**0.046**	**0.273**	**0.522**	-
*Planigale* sp. 1			Chichester	Fortescue	Hamersley	Roebourne
	94	Chichester	-			
	4	Fortescue	**0.174**	-		
	76	Hamersley	**0.064**	**0.221**	-	
	10	Roebourne	0.041	**0.173**	**0.081**	-
*Planigale* sp. 2			Chichester	Fortescue	Hamersley	Roebourne
	13	Chichester	-			
	6	Fortescue	**0.372**	-		
	14	Hamersley	0.035	**0.402**	-	
	6	Roebourne	**0.263**	**0.231**	**0.344**	-

Bold values indicate *p* < 0.05.

**Table 4:** Expansion statistics for the concatenated mtDNA (*CR* + *cytb*) and the phased nuclear (*ω-globin*) gene alignments of the six species in this study. Some species were grouped according to mtDNA haplo groups from the network analysis that also successfully converged in the skyline analysis. Number of samples (*n*), sequence length in base pairs excluding alignment gaps (L), Tajima’s D (*D*), Ramos-Onsins and Rozas R2 (*R2*), Fu’s Fs (*Fs*). Significant test scores are shown in bold font (*p* values of <0.01 considered significant for *Fs*).

Species	*n*	L	*D*	*R2*	*Fs*
**Concatenated mtDNA**					
*S. ooldea* (all)	70	1625	−0.223	0.093	**−0.114**
Haplo group 1	15	1625	−0.080	0.127	−0.022
Haplo group 2 and 3	55	1625	−1.163	0.065	**−25.257**
*S. youngsoni* (all)	113	1923	−0.944	0.064	**−0.092**
Haplo group 1	66	1923	−1.096	0.067	−12.086
Haplo group 2	26	1923	−1.474	**0.063**	**−7.678**
*S. macroura* (WA)	177	1589	−1.395	**0.046**	**−81.38**
*S. longicaudata*	30	1369	−1.446	**0.068**	**−12.01**
*Planigale* sp. 1 (all)	206	1609	−1.649	**0.038**	**−130.77**
Haplo group 1	96	1609	**−1.890**	**0.037**	**−59.99**
Haplo group 2	94	1609	**−2.003**	**0.034**	**−48.80**
*Planigale* sp. 2	39	1391	−1.676	**0.054**	**−20.21**
**Phased *ω-Globin***					
*S. ooldea*	122	774	0.657	0.119	−4.43
*S. youngsoni*	276	798	−1.429	**0.037**	**−36.63**
*S. macroura* (WA)	404	787	−1.640	**0.019**	−9.81
*S. longicaudata*	112	777	−0.069	0.092	−2.62
*Planigale* sp. 1	432	777	−0.894	0.048	**−11.94**
*Planigale* sp. 2	90	788	−1.623	0.060	−5.49

Significant test scores are shown in bold font (*p* values of <0.01 considered significant for *Fs*).

## Supplementary Materials

The following are available online at https://www.mdpi.com/2073-4425/11/9/963/s1, Figure S1: Nuclear DNA networks, Figure S2: Concatenated mtDNA networks and maps showing the geographic distribution of major haplo groups, Table S1: Genbank Accession numbers and specimen voucher information for samples sequenced, Table S2: Primers used for PCR amplification and sequencing.

## References

[1] Hewitt G. (2000). The genetic legacy of the Quaternary ice ages. Nature.

[2] Galbreath K.E., Hafner D.J., Zamudio K.R. (2009). When cold is better: Climate-driven elevation shifts yeild complex patterns of diversification and demography in an alpine specialist (American pika, *Ochotona princeps*). Evol. Int. J. Org. Evol..

[3] Pedreschi D., García-Rodríguez O., Yannic G., Cantarello E., Diaz A., Golicher D., Korstjens A.H., Heckel G., Searle J.B., Gillingham P. (2019). Challenging the European southern refugium hypothesis: Species-specific structures versus general patterns of genetic diversity and differentiation among small mammals. Glob. Ecol. Biogeogr..

[4] Velo-Antón G., Martínez-Freiría F., Pereira P., Crochet P.-A., Brito J.C. (2018). Living on the edge: Ecological and genetic connectivity of the spiny-footed lizard, *Acanthodactylus aureus*, confirms the Atlantic Sahara desert as a biogeographic corridor and centre of lineage diversification. J. Biogeogr..

[5] Byrne M., Yeates D.K., Joseph L., Kearney M., Bowler J., Williams M.A.J., Cooper S., Donnellan S.C., Keogh J.S., Leys R. (2008). Birth of a biome: Insights into the assembly and maintenance of the Australian arid zone biota. Mol. Ecol..

[6] Kearns A.M., Joseph L., Toon A., Cook L.G. (2014). Australia’s arid-adapted butcherbirds experienced range expansions during Pleistocene glacial maxima. Nat. Commun..

[7] Stern H., De Hoedt G., Ernst J. (2000). Objective classification of Australian climates. Aust. Meteorol. Mag..

[8] McGowran B., Holdgate G.R., Li Q., Gallagher S.J. (2004). Cenozoic stratigraphic succession in southeastern Australia. Aust. J. Earth Sci..

[9] Byrne M., Steane D.A., Joseph L., Yeates D.K., Jordan G.J., Crayn D., Aplin K., Cantrill D.J., Cook L.G., Crisp M.D. (2011). Decline of a biome: Evolution, contraction, fragmentation, extinction and invasion of the Australian mesic zone biota. J. Biogeogr..

[10] Williams M., Dunkerley D., De Deckker P., Kershaw P., Chappell J. (1998). Quaternary Environments.

[11] Fujita M.K., McGuire J.A., Donnellan S.C., Moritz C. (2010). Diversification and persistence at the arid-monsoonal interface: Australia-wide biogeography of the Bynoe’s gecko (*Heteronotia binoei*; Gekkonidae). Evol. Int. J. Org. Evol..

[12] Ford F., Johnson C. (2007). Eroding abodes and vanished bridges: Historical biogeography of the substrate specialist pebble-mound mice (*Pseudomys*). J. Biogeogr..

[13] Neaves L.E., Zenger K.R., Prince R.I.T., Eldridge M.D.B. (2012). Impact of Pleistocene aridity oscillations on the population history of a widespread, vagile Australian mammal, *Macropus fuliginosus*. J. Biogeogr..

[14] Pepper M., Ho S.Y.W., Fujita M.K., Scott Keogh J. (2011). The genetic legacy of aridification: Climate cycling fostered lizard diversification in Australian montane refugia and left low-lying deserts genetically depauperate. Mol. Phylogenet. Evol..

[15] Joseph L., Wilke T. (2007). Lack of phylogeographic structure in three widespread Australian birds reinforces emerging challenges in Australian historical biogeography. J. Biogeogr..

[16] Wadley J.J., Fordham D.A., Thomson V.A., Ritchie E.G., Austin J.J. (2016). Phylogeography of the antilopine wallaroo (*Macropus antilopinus*) across tropical northern Australia. Ecol. Evol..

[17] McKenzie N.L., Van Leeuwen S., Pinder A.M. (2009). Introduction to the Pilbara Biodiversity Survey, 2002–2007. Rec. West. Aust. Mus. Suppl..

[18] Cracraft J. (1991). Patterns of diversification within continental biotas: Hierarchical congruence among the areas of endemism of Australian vertebrates. Aust. Syst. Bot..

[19] Doughty P., Rolfe J.K., Burbidge A.H., Pearson D.J., Kendrick P.G. (2011). Herpetological assemblages of the Pilbara biogeographic region, Western Australia: Ecological associations, biogeographic patterns and conservation. Rec. West. Aust. Mus. Suppl..

[20] Departement of Environment and Energy (2012). Interim Biogeographic Regionalisation for Australia v. 7 (IBRA).

[21] Byrne M., Millar M.A., Coates D.J., Macdonald B.M., McArthur S.M., Zhou M., van Leeuwen S. (2017). Refining expectations for environmental characteristics of refugia: Two ranges of differing elevation and topographical complexity are mesic refugia in an arid landscape. J. Biogeogr..

[22] Pepper M., Doughty P., Keogh J.S. (2013). Geodiversity and endemism in the iconic Australian Pilbara region: A review of landscape evolution and biotic response in an ancient refugium. J. Biogeogr..

[23] Anderson B.M., Barrett M.D., Krauss S.L., Thiele K. (2016). Untangling a species complex of arid zone grasses (*Triodia*) reveals patterns congruent with co-occurring animals. Mol. Phylogenet. Evol..

[24] Shoo L.P., Rose R., Doughty P., Austin J.J., Melville J. (2008). Diversification patterns of pebble-mimic dragons are consistent with historical disruption of important habitat corridors in arid Australia. Mol. Phylogenet. Evol..

[25] Woinarski J.C.Z., Burbidge A.A., Harrison P.L. (2015). Ongoing unraveling of a continental fauna: Decline and extinction of Australian mammals since European settlement. Proc. Natl. Acad. Sci. USA.

[26] Crowther M.S., Blacket M.J., Jones M., Dickman C.R., Archer M. (2003). Biogeography and speciation in the Dasyuridae: Why are there so many kinds of dasyurids. Predators with Pouches: The Biology of Carnivorous Marsupials.

[27] Mitchell K.J., Pratt R.C., Watson L.N., Gibb G.C., Llamas B., Kasper M., Edson J., Hopwood B., Male D., Armstrong K.N. (2014). Molecular phylogeny, biogeography, and habitat preference evolution of marsupials. Mol. Biol. Evol..

[28] Haythornthwaite A.S., Dickman C.R. (2006). Long-distance movements by a small carnivorous marsupial: How *Sminthopsis youngsoni* (Marsupialia: Dasyuridae) uses habitat in an Australian sandridge desert. J. Zool..

[29] Krajewski C., Woolley P.A., Westerman M. (2000). The evolution of reproductive strategies in dasyurid marsupials: Implications of molecular phylogeny. Biol. J. Linn. Soc..

[30] Morton S.R., Archer M. (1982). Dasyurid marsupials of the Australian arid zone: An ecological review. Carnivorous Marsupials Volume 1.

[31] Geiser F. (1994). Hibernation and daily dorpor in marsupials—A review. Aust. J. Zool..

[32] Van Dyck S., Strahan R., Van Dyck S., Strahan R. (2008). The Mammals of Australia.

[33] Spencer W. (1909). Description of a new species of *Sminthopsis*. Proc. R. Soc. Vic..

[34] Gould J. (1845). Descriptions of five new species of mammals. Proc. Zool. Soc. Lond..

[35] Troughton E. (1965). A review of the marsupial genus *Sminthopsis* (Phascogalinae) and diagnoses of new forms. Proc. Linn. Soc. N.S.W..

[36] McKenzie N.L., Archer M. (1982). *Sminthopsis youngsoni* (Marsupialia: Dasyuridae) the Lesser Hairy-footed Dunnart, a new species from arid Australia. Aust. Mammal..

[37] Westerman M., Blacket M.J., Hintz A., Armstrong K., Woolley P.A., Krajewski C. (2016). A plethora of planigales: Genetic variability and cryptic species in a genus of dasyurid marsupials from northern Australia. Aust. J. Zool..

[38] Archer M. (1981). Revision of the dasyurid marsupial genus *Sminthopsis* Thomas. Bull. Am. Mus. Nat. Hist..

[39] Blacket M.J., Adams M., Cooper S.J.B., Krajewski C., Westerman M. (2001). Systematics and evolution of the dasyurid marsupial genus *Sminthopsis*: I. the Macroura species group. J. Mamm. Evol..

[40] Gibson L.A., Mckenzie N.L. (2009). Environmental associations of small ground-dwelling mammals in the Pilbara region, Western Australia. Rec. West. Aust. Mus. Suppl..

[41] Hess P.P. (2010). The Australian desert dunefields: Formation and evolution in an old, flat, dry continent. Geol. Soc. Lond. Spec. Publ..

[42] Umbrello L.S., Woolley P.A., Westerman M. (2017). Species relationships in the dasyurid marsupial genus *Pseudantechinus* (Marsupialia: Dasyuridae): A re-examination of the taxonomic status of *Pseudantechinus Roryi*. Aust. J. Zool..

[43] Krajewski C., Blacket M., Buckley L., Westerman M. (1997). A multigene assessment of phylogenetic relationships within the dasyurid marsupial subfamily Sminthopsinae. Mol. Phylogenet. Evol..

[44] Blacket M.J., Krajewski C., Labrinidis A., Cambron B., Cooper S., Westerman M. (1999). Systematic relationships within the dasyurid marsupial tribe Sminthopsini—A multigene approach. Mol. Phylogenet. Evol..

[45] Kearse M., Moir R., Wilson A., Stones-Havas S., Cheung M., Sturrock S., Buxton S., Cooper A., Markowitz S., Duran C. (2012). Geneious Basic: An integrated and extendable desktop software platform for the organization and analysis of sequence data. Bioinformatics.

[46] Stamatakis A. (2006). RAxML-VI-HPC: Maximum likelihood-based phylogenetic analyses with thousands of taxa and mixed models. Bioinformatics.

[47] Lanfear R., Calcott B., Ho S.Y.W., Guindon S. (2012). PartitionFinder: Combined selection of partitioning schemes and substitution models for phylogenetic analyses. Mol. Biol. Evol..

[48] Leigh J.W., Bryant D. (2015). PopART: Full-feature software for haplotype network construction. Methods Ecol. Evol..

[49] Castresana J. (2000). Selection of conserved blocks from multiple alignments for their use in phylogenetic analysis. Mol. Biol. Evol..

[50] Dereeper A., Guignon V., Blanc G., Audic S., Buffet S., Chevenet F., Dufayard J.-F., Guindon S., Lefort V., Lescot M. (2008). Phylogeny.fr: Robust phylogenetic analysis for the non-specialist. Nucleic Acids Res..

[51] Stephens M., Smith N.J., Donnelly P. (2001). A new statistical method for haplotype reconstruction from population data. Am. J. Hum. Genet..

[52] Flot J.-F. (2010). seqPHASE: A web tool for interconverting PHASE input/output files and FASTA sequence alignments. Mol. Ecol. Resour..

[53] Clement M., Posada D., Crandall K.A. (2000). TCS: A computer program to estimate gene genealogies. Mol. Ecol..

[54] Excoffier L., Lischer H.E.L. (2010). Arlequin suite ver 3.5: A new series of programs to perform population genetics analyses under Linux and Windows. Mol. Ecol. Resour..

[55] Kalinowski S.T. (2005). HP-RARE 1.0: A computer program for performing rarefaction on measures of allelic richness. Mol. Ecol. Notes.

[56] Librado P., Rozas J. (2009). DnaSP v5: A software for comprehensive analysis of DNA polymorphism data. Bioinformatics.

[57] Heled J., Drummond A.J. (2008). Bayesian inference of population size history from multiple loci. BMC Evol. Biol..

[58] Bouckaert R., Heled J., Kühnert D., Vaughan T., Wu C.-H., Xie D., Suchard M.A., Rambaut A., Drummond A.J. (2014). BEAST 2: A software platform for Bayesian evolutionary analysis. PLoS Comput. Biol..

[59] Ho S.Y.W., Shapiro B. (2011). Skyline-plot methods for estimating demographic history from nucleotide sequences. Mol. Ecol. Resour..

[60] Jia F., Lo N., Ho S.Y.W. (2014). The impact of modelling rate heterogeneity among sites on phylogenetic estimates of intraspecific evolutionary rates and timescales. PLoS ONE.

[61] Krajewski C., Wroe S., Westerman M. (2000). Molecular evidence for the pattern and timing of cladogenesis in dasyurid marsupials. Zool. J. Linn. Soc..

[62] Bennison K., Dickman C.R., Godfree R. (2013). Habitat use and ecological observations of the Ooldea dunnart (*Sminthopsis ooldea*) at Uluru–Kata Tjuta National Park, Northern Territory. Aust. Mammal..

[63] Ford J. (1987). Hybrid zones in Australian birds. Emu Austral. Ornithol..

[64] Pepper M., Doughty P., Keogh J.S. (2006). Molecular phylogeny and phylogeography of the Australian *Diplodactylus stenodactylus* (Gekkota; Reptilia) species-group based on mitochondrial and nuclear genes reveals an ancient split between Pilbara and non-Pilbara *D. stenodactylus*. Mol. Phylogenet. Evol..

[65] Fujioka T., Chappell J. (2010). History of Australian aridity: Chronology in the evolution of arid landscapes. Geol. Soc. London Spec. Publ..

[66] McLean A.L., Cooper S.J.B., Lancaster M.L., Gaikhorst G., Lambert C., Moseby K., Read J., Ward M., Carthew S.M. (2018). Small marsupial, big dispersal? Broad—And fine-scale genetic structure of an endangered marsupial from the Australian arid zone. Aust. J. Zool..

[67] Oliver P.M., Couper P.J., Pepper M. (2014). Independent transitions between monsoonal and arid biomes revealed by systematic revison of a complex of Australian geckos (*Diplodactylus*; Diplodactylidae). PLoS ONE.

[68] Levy E., Byrne M., Huey J.A., Hillyer M.J., Firman R.C., Ottewell K.M. (2019). Limited influence of landscape on the genetic structure of three small mammals in a heterogeneous arid environment. J. Biogeogr..

[69] Hayes G. (2018). Living with fire: Ecology and genetics of the dasyurid marsupial *Dasykaluta rosamondae*. Ph.D. Thesis.

[70] Spencer P.B.S., How R.A., Hillyer M., Cook A., Morris K., Stevenson C.A., Umbrello L.S. (2013). Genetic Analysis of Northern Quolls from the Pilbara Region of Western Australia. Year One—Final Report.

[71] Schmitt L.H., Bradley A.J., Kemper C.M., Kitchener D.J., Humphreys W.F., How R.A. (1989). Ecology and physiology of the northern quoll, *Dasyurus hallucatus* (Marsupialia, Dasyuridae), at Mitchell Plateau, Kimberley, Western Australia. J. Zool..

[72] Eldridge M.D.B., Potter S., Johnson C.N., Ritchie E.G. (2014). Differing impact of a major biogeographic barrier on genetic structure in two large kangaroos from the monsoon tropics of Northern Australia. Ecol. Evol..

[73] Strasburg J.L., Kearney M., Moritz C., Templeton A.R. (2007). Combining phylogeography with distribution modeling: Multiple pleistocene range expansions in a parthenogenetic gecko from the Australian arid zone. PLoS ONE.

[74] Anderson B.M., Thiele K.R., Grierson P.F., Krauss S.L., Nevill P.G., Small I.D., Zhong X., Barrett M.D. (2019). Recent range expansion in Australian hummock grasses (*Triodia*) inferred using genotyping-by-sequencing. AoB Plants.

[75] Woolley P.A., Krajewski C., Westerman M. (2015). Phylogenetic relationships within *Dasyurus* (Dasyuromorphia: Dasyuridae): Quoll systematics based on molecular evidence and male characteristics. J. Mammal..

[76] Moritz C.C. (1994). Defining “Evolutionarily Significant Units” for conservation. Trends Ecol. Evol..

[77] Dickman C.R., Jones M., Dickman C., Archer M. (2003). Distributional ecology of dasyurid marsupials. Predators with Pouches: The Biology of Carnivorous Marsupials.

[78] Bino G., Ramp D., Kingsford R.T. (2013). Niche evolution in Australian terrestrial mammals? Clarifying scale-dependencies in phylogenetic and functional drivers of co-occurrence. Evol. Ecol..

[79] García-Navas V., Rodríguez-Rey M., Westerman M. (2018). Bursts of morphological and lineage diversification in modern dasyurids, a ‘classic’ adaptive radiation. Biol. J. Linn. Soc..

[80] García-Navas V., Kear B.P., Westerman M. (2020). The geography of speciation in dasyurid marsupials. J. Biogeogr..

[81] Byrne M., Joseph L., Yeates D.K., Roberts J.D., Edwards D. (2018). Evolutionary history. On the Ecology of Australia’s Arid Zone.

