# Supplementary material for: Multi-Species Phylogeography of Arid-Zone Sminthopsinae (Marsupialia: Dasyuridae) Reveals Evidence of Refugia and Population Expansion in Response to Quaternary Change
Source: Genes (Basel). 2020 Aug 20;11(9):963. doi: 10.3390/genes11090963 (PMC7563968; doi:10.3390/genes11090963)
Supplement: Supplementary file 1 [file genes-11-00963-s001.zip › Supplementary material/Supplementary Material.docx]

**Supplementary Material for Umbrello et al. 2020 Multi-species phylogeography of arid-zone Sminthopsinae (Marsupialia: Dasyuridae) reveals evidence of refugia and population expansion in response to Quaternary change.**

**Table S2:** Primers used for PCR amplification and sequencing

| Name [ref] | Direction | Sequence |
| --- | --- | --- |
|  |  | Control region (left domain) |
| L15999M[1] | FWD | 5'-ACCATCAACACCCAAAGCTGA-3' |
| H16498M[1] | REV | 5'-CCTGAAGTAGCAACCAGTAG-3' |
|  |  | ω-globin intron 2 |
| G314[2] | FWD | 5'-GGAATCATGGCAAGAAGGTG-3' |
| G424[2] | REV | 5'-CCGGAGGTGTTYAGTGGTATTTTC-3' |
|  |  | Cytochrome b |
| MVZ-05[3] | FWD | 5'-CGAAGCTTGATATGAAAAACCATCGTTG-3' |
| MVZ-16[3] | REV | 5'-AAATAGGAARTATCAYTCTGGTTTRAT-3' |
| L15311[4] | FWD | 5'-CTACCATGAGGACAAATATC-3' |
| L15656[4] | FWD | 5'-AACCTACTAGGAGACCCAGA-3' |
| CytBf[5] | FWD | 5'-TGAGGTGCAACAGTNATTAC-3' |
| H15149[6] | REV | 5'-AAACTGCAGCCCCTCAGAATGATATTTGTCCTCA-3' |
| H15767[7] | REV | 5'-ATGAAGGGATGTTCTACTGGTTG-3' |
| H15915A[8] | REV | 5'-AACCTTCGTTGTTGGCTTACAAGAC-3' |

To obtain the complete *cytb* gene (1146 bp) three fragments were amplified using primer pairs; MVZ-05/H15149, L15131/H15767 and L15656/H15915, giving reads of about 450 bp, 620 bp and 400 bp respectively.

**References**

1. Fumagalli, L.; Pope, L.C.; Taberlet, P.; Moritz, C. Versatile primers for the amplification of the mitochondrial DNA control region in marsupials. *Mol. Ecol.* **1997**, *6*, 1199–1201.

2. Wheeler, D.; Hope, R.; Cooper, S.B.; Dolman, G.; Webb, G.C.; Bottema, C.D.; Gooley, A.A.; Goodman, M.; Holland, R.A. An orphaned mammalian beta-globin gene of ancient evolutionary origin. *Proc. Natl. Acad. Sci. U. S. A.* **2001**, *98*, 1101–6.

3. Da Silva, M.N.F.; Patton, J.L. Amazonian Phylogeography: mtDNA Sequence Variation in Arboreal Echimyid Rodents (Caviomorpha). *Mol. Phylogenet. Evol.* **1993**, *2*, 243–255.

4. Helm-Bychowski, K.; Cracraft, J. Recovering phylogenetic signal from DNA sequences: relationships within the corvine assemblage (class aves) as inferred from complete sequences of the mitochondrial DNA cytochrome-b gene. *Mol. Biol. Evol.* **1993**, *10*, 1196–214.

5. Nekola, J.C.; Coles, B.F.; Horsák, M. Species assignment in *Pupilla* (Gastropoda: Pulmonata: Pupillidae): integration of DNA-sequence data and conchology. *J. Molluscan Stud.* **2015**, *81*, 196–216.

6. Kocher, T.D.; Thomas, W.K.; Meyer, A.; Edwards, S. V; Pääbo, S.; Villablanca, F.X.; Wilson, A.C. Dynamics of mitochondrial DNA evolution in animals: amplification and sequencing with conserved primers. *Proc. Natl. Acad. Sci. U. S. A.* **1989**, *86*, 6196–200.

7. Edwards, S. V.; Arctander, P.; Wilson, A.C. Mitochondrial resolution of a deep branch in the genealogical tree for perching birds. *Proc. R. Soc. B Biol. Sci.* **1991**, *243*, 99–107.

8. Krajewski, C.; Blacket, M.; Buckley, L.; Westerman, M. A multigene assessment of phylogenetic relationships within the dasyurid marsupial subfamily Sminthopsinae. *Mol. Phylogenet. Evol.* **1997**, *8*, 236–248, doi:10.1006/mpev.1997.0421.


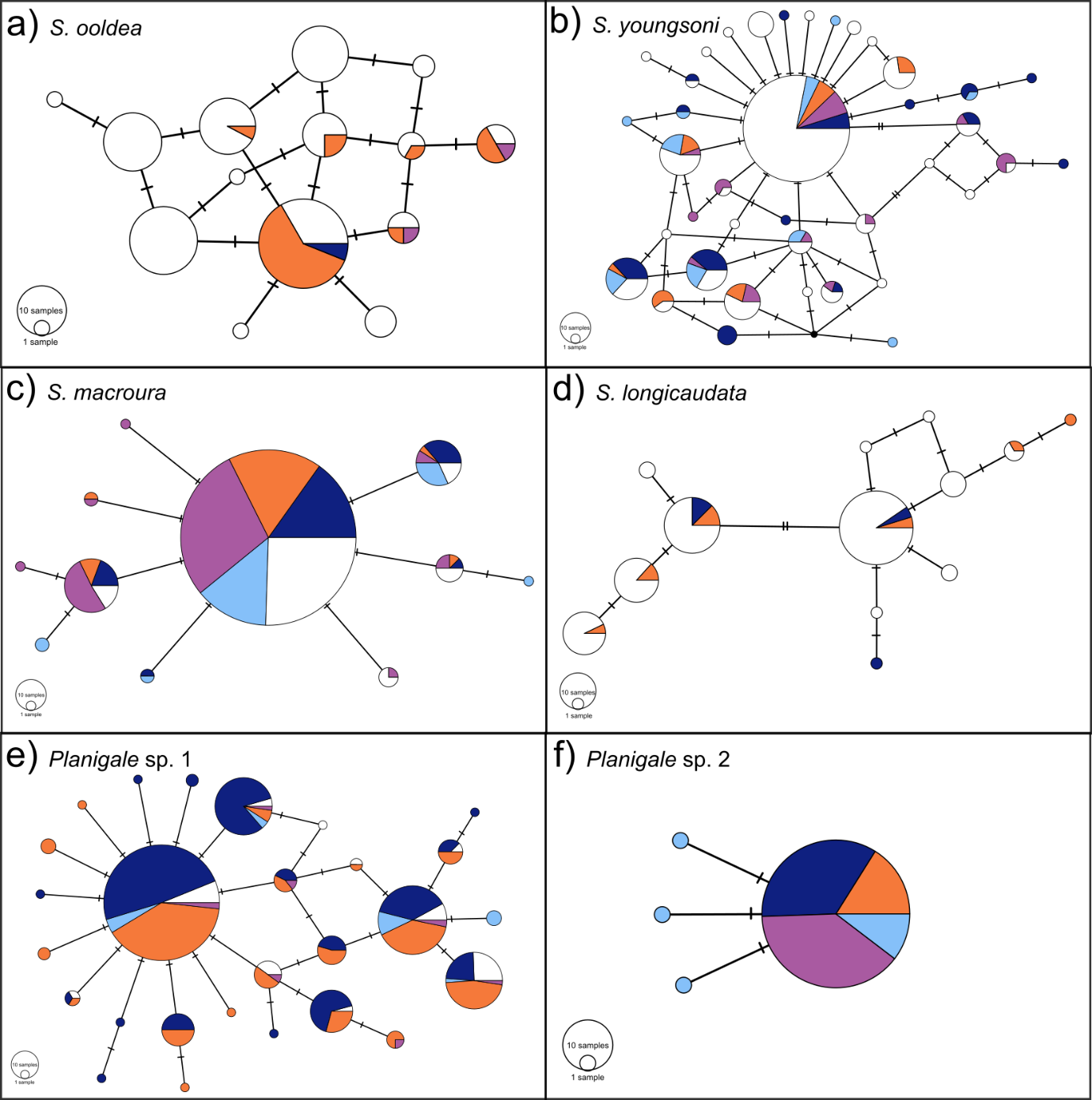


**Figure S1:** Nuclear DNA networks for a) *Sminthopsis ooldea*, b) *S. youngsoni*, c) *S. macroura*, d) *S. longicaudata*, e) *Planigale* sp. 1 and f) *Planigale* sp. 2. Colours represent IBRA subregions (see Figure 3 in the main text). Circle size indicates the number of individuals sharing that haplotype, with larger circles indicating more common haplotypes, hatch-marks show missing haplotypes.


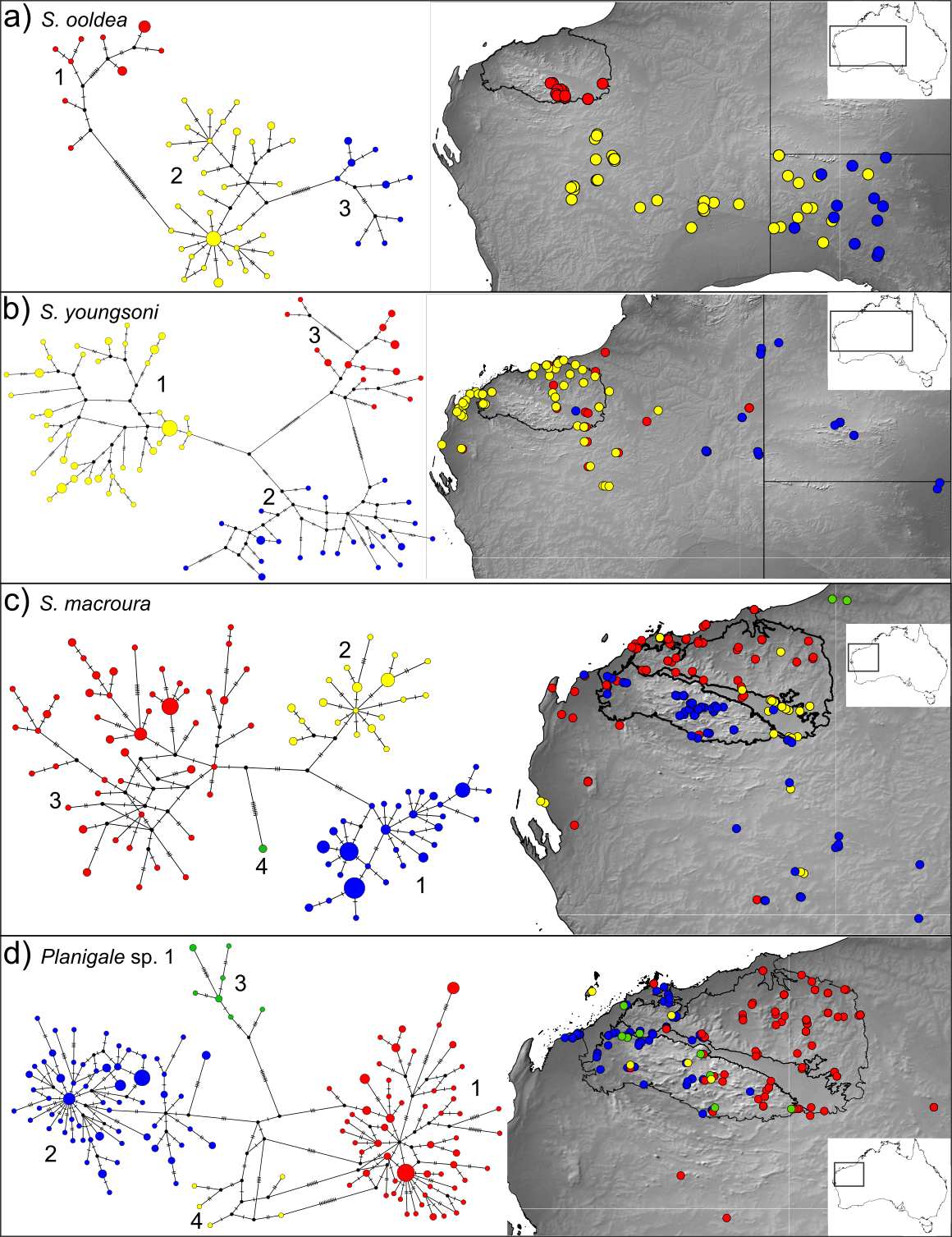


**Figure S2:** Concatenated mtDNA networks and map of samples for **a)** *Sminthopsis ooldea* (n=70), **b)** *S. youngsoni* (n=113), **c)** *S. macroura* (n=177) and **d)** *Planigale* sp. 1 (n=206), major haplo-groups are coloured for emphasis and numbered, circle size indicates the number of individuals sharing haplotypes, with larger circles indicating more common haplotypes, small black circles indicate nodes, hatch-marks show missing haplotypes. Maps show Pilbara bioregion and IBRA subregions and colours of samples in maps correspond to the networks.
